# Interaction of Apoptotic Cells with Macrophages Upregulates COX-2/PGE_2_ and HGF Expression via a Positive Feedback Loop

**DOI:** 10.1155/2014/463524

**Published:** 2014-05-15

**Authors:** Ji Yeon Byun, Young-So Youn, Ye-Ji Lee, Youn-Hee Choi, So-Yeon Woo, Jihee Lee Kang

**Affiliations:** ^1^Department of Physiology, School of Medicine, Ewha Womans University, 911-1 Mok-6-dong, Yangcheon-gu, Seoul 158-710, Republic of Korea; ^2^Tissue Injury Defense Research Center, School of Medicine, Ewha Womans University, 911-1 Mok-6-dong, Yangcheon-gu, Seoul 158-710, Republic of Korea; ^3^Global Top 5 Research Program, Ewha Womans University, Seoul 158-710, Republic of Korea; ^4^Department of Microbiology, School of Medicine, Ewha Womans University, Seoul 158-710, Republic of Korea

## Abstract

Recognition of apoptotic cells by macrophages is crucial for resolution of inflammation, immune tolerance, and tissue repair. Cyclooxygenase-2 (COX-2)/prostaglandin E2 (PGE_2_) and hepatocyte growth factor (HGF) play important roles in the tissue repair process. We investigated the characteristics of macrophage COX-2 and PGE_2_ expression mediated by apoptotic cells and then determined how macrophages exposed to apoptotic cells *in vitro* and *in vivo* orchestrate the interaction between COX-2/PGE_2_ and HGF signaling pathways. Exposure of RAW 264.7 cells and primary peritoneal macrophages to apoptotic cells resulted in induction of COX-2 and PGE_2_. The COX-2 inhibitor NS-398 suppressed apoptotic cell-induced PGE_2_ production. Both NS-398 and COX-2-siRNA, as well as the PGE_2_ receptor EP2 antagonist, blocked HGF expression in response to apoptotic cells. In addition, the HGF receptor antagonist suppressed increases in COX-2 and PGE_2_ induction. The *in vivo* relevance of the interaction between the COX-2/PGE_2_ and HGF pathways through a positive feedback loop was shown in cultured alveolar macrophages following *in vivo* exposure of bleomycin-stimulated lungs to apoptotic cells. Our results demonstrate that upregulation of the COX-2/PGE_2_ and HGF in macrophages following exposure to apoptotic cells represents a mechanism for mediating the anti-inflammatory and antifibrotic consequences of apoptotic cell recognition.

## 1. Introduction

The clearance of apoptotic cells by tissue macrophages and nonprofessional phagocytes is an essential process in tissue homeostasis, immunity, and resolution of inflammation. Apoptotic cell recognition actively leads to the production of anti-inflammatory mediators such as TGF-*β*, IL-10, and PGE_2_ [1, 2]. Interactions between apoptotic and phagocytic cells play important roles in the regeneration and repair of damaged tissues by induction of growth-maintenance factors, such as VEGF, HGF, and PGE_2_, which can reconstitute the damaged tissue leading to decrease in fibroproliferative sequelae [3, 4].

The COX-2 enzymatic product, PGE_2_, is a lipid mediator that, similar to TGF-*β*, has been shown to have pro- or anti-inflammatory properties under differing circumstances. In the lung, PGE_2_ plays roles in tissue repair processes and in limiting immune-inflammatory responses [5]. PGE_2_, which is generated via the conversion of arachidonic acid to PGH_2_ via the COX-1 or COX-2 enzymes, is the major eicosanoid produced by lung fibroblasts and many other lung cells, including alveolar macrophages. Through E-prostanoid receptor 2 (EP2)-mediated increases in intracellular cyclic AMP, PGE_2_ directly inhibits several major pathobiologic functions of fibroblasts, including chemotaxis, proliferation [6], collagen synthesis [7], and differentiation into myofibroblasts [8]. Diminished PGE_2_ production and/or signaling can be observed in human and animal lung fibrosis [9, 10].

In studies by Fadok and colleagues [1], the exogenous addition of PGE_2_ to LPS-stimulated macrophages decreased their production of proinflammatory cytokines, such as TNF-*α*, IL-1*β*, and IL-8. Addition of indomethacin restored suppression of proinflammatory cytokine production caused by apoptotic cells in macrophages stimulated with LPS. Recently, we demonstrated* in vitro* that apoptotic cell-induced HGF reduces inflammatory cytokine expression in macrophages [11]. Moreover, we found that* in vivo *exposure to apoptotic cells induces anti-inflammatory effects through induction of COX-2/PGE_2_, and HGF signaling in bleomycin-stimulated lungs, using pharmacologic approaches [11, 12]. Our previous studies also demonstrated that* in vivo* exposure to apoptotic cells resulted in enhanced expression of HGF [11] and COX-2 and secretion of PGE_2_ [12] until the late fibrotic phase in bleomycin-induced lung injury. These data indicate that the anti-inflammatory and antifibrotic effects in the lung following apoptotic cell instillation are correlated with coordinated increases in HGF and COX-2/PGE_2_ signaling. However, the mechanism underlying the prolonged induction of HGF and COX-2 by apoptotic cells is not clearly understood at the cellular model* in vitro*. Therefore, a key issue is whether recognition of apoptotic cells by macrophages can direct the signaling pathways toward coordinated induction of these anti-inflammatory and growth-maintenance factors. In the present study, we first characterized induction of COX-2 and PGE_2_ by* in vitro* exposure of RAW 264.7 cells and murine primary peritoneal macrophages to apoptotic cells. We then determined how macrophages programmed by apoptotic cells orchestrate the interaction between COX-2/PGE_2_ and HGF signaling.

## 2. Materials and Methods 

### 2.1. Reagents

Actinomycin D, cycloheximide, and indomethacin were purchased from Sigma-Aldrich (St. Louis, MO), and NS-398, AH-6809, GW-627368X, and PGE_2_ were purchased from Cayman Chemical (Ann Arbor, MI). PHA-665752 was obtained from Santa Cruz Biotechnology (Santa Cruz, CA). The gene-specific relative RT-PCR kit was obtained from Invitrogen (Carlsbad, CA), and M-MLV reverse transcriptase was purchased from Enzynomics (Hanam, Korea). ELISA kits for HGF and TGF-*β*1 were obtained from R&D Systems, and the enzyme immunoassay (EIA) kits for PGE_2_ and 15-deoxy-Δ12, 14-PG J_2_ (15d-PGJ_2_) were obtained from Assay Designs (Ann Arbor, MI). The antibodies used in this study were against COX-1 and COX-2 (Cayman Chemical), HGF-*α* (Santa Cruz Biotechnology), and *β*-actin (Sigma-Aldrich).

### 2.2. Cell Lines, Culture, and Stimulation

Murine RAW 264.7 macrophages (American Type Culture Collection, Rockville, MD) were plated at 3 × 10^5^ cells/mL and incubated overnight in Dulbecco's modified Eagle's medium (DMEM, Media Tech Inc., Washington, DC) supplemented with 10% heat-inactivated FBS, 2 mM L-glutamine, 100 U/mL penicillin, and 100 *μ*g/mL streptomycin at 37°C and 5% CO_2_. Before stimulation, the medium was replaced with serum-free X-vivo 10. The macrophages were stimulated with apoptotic or viable cells (1.5 × 10^6^ cells/mL).

### 2.3. Isolation and Culture of Primary Cells

Resident peritoneal macrophages were isolated from 6- to 7-week-old pathogen-free male C57BL/6 mice (Orient Bio, Sungnam, Korea) weighing 20–22 g. The Animal Care Committee of the Ewha Medical Research Institute (Seoul, Republic of Korea) approved the experimental protocol (number 11-0171). Resident peritoneal macrophages were isolated by lavage with 5 mL of ice-cold sterile HBSS after mice were euthanized with CO_2_. The lavage fluid was centrifuged and resident peritoneal cells were plated at 1 × 10^6^ cells/well and cultured in a humidified 5% CO_2_ atmosphere at 37°C in DMEM supplemented with 10% heat-inactivated FBS, 2 mM l-glutamine, 100 *μ*g/mL of streptomycin, and 100 U/mL of penicillin. The isolated macrophages were stimulated with apoptotic Jurkat T cells (3 × 10^6^ cells/mL). Suspended peritoneal macrophages were over 95% viable, as determined by trypan blue dye exclusion. Individual thymocytes were isolated from 3- to 4-week-old mice by mincing the thymus through a 70 *μ*m pore size cell strainer (BD Biosciences, Bedford, MA). Human neutrophils were obtained from normal, healthy donors in accordance with a protocol reviewed and approved by the Institutional Review Board. Using endotoxin-free reagents and plastic ware, human neutrophils were isolated by the plasma Percoll method previously [13].

### 2.4. Induction of Apoptosis

Human Jurkat T lymphocytes, HeLa epithelial cells, and murine thymocytes were exposed to UV irradiation at 254 nm for 10 min followed by incubation in RPMI-1640 with 10% fetal bovine serum for 2 h at 37°C and 5% CO_2_. Human neutrophils (>95% purity) were either cultured overnight at 9 × 10^5^/mL in RPMI 1640 at 37°C in 5% CO_2_ or UV-irradiated for 10 min followed by incubation for 2 h before addition to macrophages (3 × 10^5^/mL). Evaluation of nuclear morphology using light microscopy on Wright-Giemsa stained samples indicated that the irradiated cells were approximately 70–80% apoptotic [14]. Apoptosis was confirmed by annexin V-FITC/propidium iodide (BD Biosciences, San Jose, CA) staining followed by flow cytometry analysis on a FACSCalibur system (BD Biosciences) [15]. Human aged neutrophils were shown to be typically 60–70% apoptotic by assessment of nuclear condensation on Wright-Giemsa stained samples and necrosis was less than 2% by trypan blue exclusion [16].

### 2.5. siRNA Transfection

RAW 264.7 cells and resident peritoneal macrophages were transiently transfected with 1 *μ*g/mL of either siRNA specifically targeting COX-2 or COX-1 or control siRNA (Bioneer, Seoul, Korea) using 5 *μ*L of siRNA transfection reagent (Genlantis, San Diego, CA) according to the manufacturer's protocol. The sequences used for COX-2 knockdown were 5′-CUA UGA UAG GAG CAU GUA A-3′ (sense) and 5′-UUA CAU GCU CCU AUC AUA G-3′ (antisense). The sequences used for COX-1 knockdown were 5′-GAG GUA GGA ACU UUG ACU A-3′ (sense) and 5′-UAG UCA AAG UUC CUA CCU C-3′ (antisense). The sequences for control siRNA were 5′-CCU ACG CCA CCA AUU UCG U-3′ (sense) and 5′-ACG AAA UUG GUG GCG UAG G-3′ (antisense). Before further experiments, cells were incubated in serum-free medium for 6 h for COX-2 siRNA or 48 h for COX-1 siRNA. For RhoA siRNA, RAW 264.7 cells were transiently transfected with 10 nM RhoA-targeting siRNA (sense: 5′-GAA GUC AAG CAU UUC UGU CTT-3′; antisense: 5′-GAC AGA AAU GCU UGA CUU CTT-3′) premixed with 6 *μ*g/mL of Lipofectin (Invitrogen). Cells were then incubated in serum-free medium for 24 h before further experimentation. None of the siRNAs used had any significant effect on cell viability.

### 2.6. RT-PCR

Total RNA was isolated from cultured cells using TRIzol reagent (Life Technologies, Carlsbad, CA). The concentration and purity of RNA were evaluated by spectrometry at 260 and 280 nm. Reverse transcription was conducted for 60 min at 42°C with 3 *μ*g of total RNA using M-MLV reverse transcriptase. The levels of COX-1, COX-2, HGF, and TGF-*β*1 mRNA were determined using a semiquantitative RT-PCR kit. The primer sequences were used as follows: mouse-specific COX-1 (sense: 5′-GGT TGA GGC ACT GGT GGA TG-3′; antisense: 5′-AGA CAG ACC CGT CAT CTC CA-3′), mouse-specific COX-2 (sense: 5′-TTC AAA AGA AGT GCT GGA AAA GGT-3′; antisense 5′-GAT CAT CTC TAC CTG AGT GTC TTT-3′), mouse-specific HGF (sense: 5′-GGA CAA GAT TGT TAT CGT GG-3′; antisense: 5′-GTT GAT CAA TCC AGT GTA GC-3′), mouse-specific TGF-*β*1 (sense: 5′-CTT CAG CTC CAC AGA GAA GAA CTG C-3′; antisense: 5′-CAC AAT CAT GTT GGA CAA CTG CTC C-3′), and mouse-specific *β*-actin (sense: 5′-GAT GAC GAT ATC GCT GCG CTG-3′; antisense 5′-GAT GAC GAT ATC GCT GCG CTG-3′). The cDNA was denatured for 5 min at 94°C and then amplified using a GeneAmp PCR System 2400 (PerkinElmer, Waltham, MA). PCR products were visualized on 1-2% agarose gels stained with GelRed. The relative fluorescence of each gene versus *β*-actin was analyzed by densitometry.

### 2.7. ELISA and EIA

Culture supernatants were collected 2–24 h after stimulation. The levels of PGE_2_ and 15d-PGJ_2_ in the supernatants were determined using EIA kits. HGF and TGF-*β*1 concentrations were measured by ELISA, according to the manufacturer's instructions.

### 2.8. Immunoblot Analysis

Cells were lysed in 0.5% Triton X-100 lysis buffer and proteins were resolved on 10% SDS-PAGE gels and then electrophoretically transferred onto nitrocellulose membranes. The membranes were blocked for 1 h at room temperature with Tris-buffered saline (100 mM Tris-Cl, pH 7.5, 150 mM NaCl, and 0.1% Tween-20) containing 5% skim milk and then incubated with various primary antibodies at 4°C overnight and probed with a mouse anti-mouse HRP-conjugated secondary antibody. Membranes were developed using an enhanced chemiluminescence system (GE Healthcare, Buckinghamshire, UK).

### 2.9. Immunocytochemistry

COX-2 protein expression was evaluated in RAW 264.7 cells by immunocytochemistry. Cells were fixed with 4% paraformaldehyde, permeabilized with Triton X-100, and stained overnight at 4°C with rabbit polyclonal anti-COX-2 antibody (1 : 400; Abcam, Cambridge, UK). Subsequently, cells were washed with PBS three times and incubated with fluorescent isothiocyanate-conjugated donkey anti-rabbit IgG (1 : 500; Jackson ImmunoResearch). The slides were mounted with Vectashield mounting medium with DAPI (Vector Laboratories, Inc.) and examined using a confocal microscope (LSM5 PASCAL; Carl Zeiss) equipped with a filter set with excitation at 488 and 543 nm.

### 2.10. Statistical Analysis

Data are expressed as the mean ± SEM. Intergroup comparisons were made using the Student's *t*-test. Statistical significance was set at a *P* value <0.05. Excel 2007 software (Microsoft, Seattle, WA) was used for statistical analyses.

## 3. Results

### 3.1. *In Vitro* Exposure of Macrophages to Apoptotic Cells Induces mRNA and Protein Expression of COX-2

**Figure 1:**
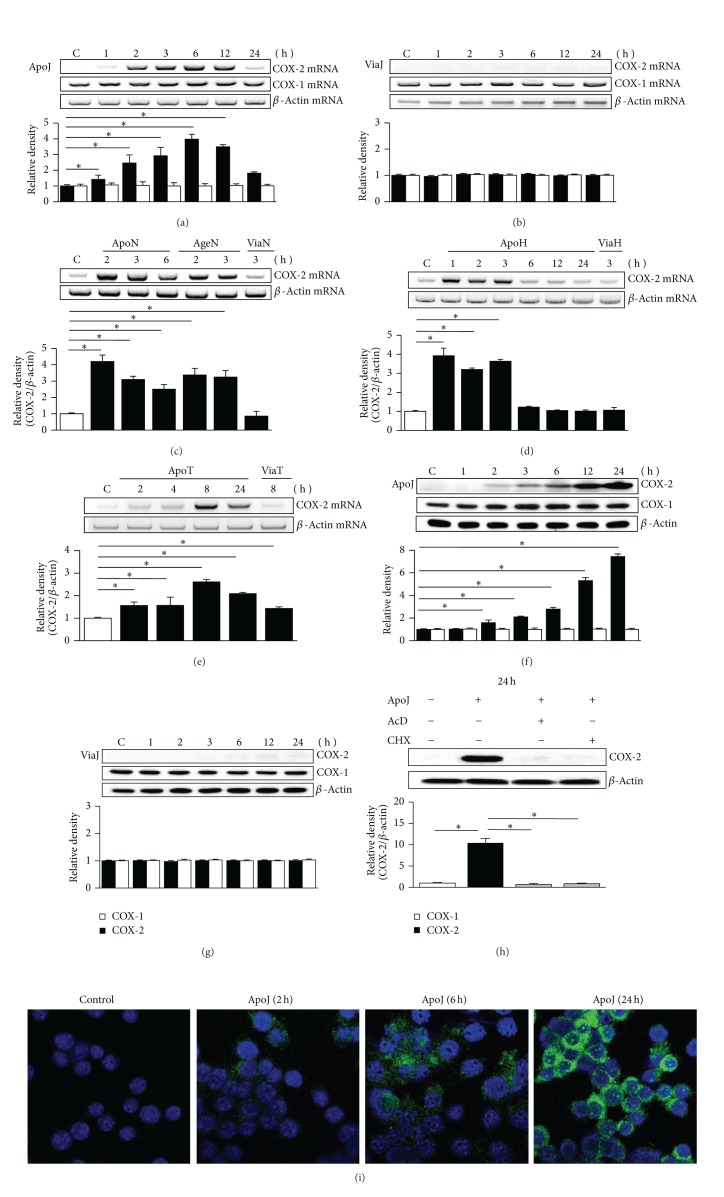
Apoptotic cells induce COX-2 expression by RAW 264.7 cells. RAW 264.7 cells were stimulated by UV-exposed apoptotic (ApoJ) or viable (ViaJ) cells of Jurkat T cells (a, b, f, g, i); UV-exposed (ApoN) or aged apoptotic (AgeN) or viable cells of neutrophils (c); UV-exposed apoptotic or viable cells of HeLa cells (ApoH, ViaH) (d); UV-exposed apoptotic or viable cells of thymocytes (ApoT, ViaT) (e) for the time indicated. (a–e) COX-2 or COX-1 mRNA levels were analyzed by semiquantitative RT-PCR and normalized to *β*-actin mRNA levels. (c) COX-2 mRNA levels were normalized to *β*-actin mRNA levels after standardization of the amount of ApoN and AgeN cells. (f–h) Immunoblots with anti-COX-2 or COX-1 antibodies were performed using cultured cell lysates. Relative values of COX-2 or COX-1 expression are indicated below the bands. (h) RAW cells were pretreated with 10 *μ*g/mL actinomycin D (AcD) or 10 *μ*g/mL cycloheximide (CHX) for 1 h before stimulation with ApoJ. Values represent mean ± SEM of three or more separate experiments; *P < 0.05. (i) Immunofluorescence staining (*green*) for COX-2 in RAW cells. Images were captured at ×800 magnification. Representative results from three separate experiments are shown.

Before evaluation of the interaction between the COX-2/PGE_2_ and HGF signaling pathways in macrophages following* in vitro* exposure to apoptotic cells, we determined the characteristics of COX-2 expression and PGE_2_ production in macrophages. First, to evaluate COX-1 and COX-2 mRNA expression, semiquantitative RT-PCR was performed using total RNA extracted from RAW 264.7 cells. COX-2 mRNA expression was distinct at 2 h after* in vitro* exposure to apoptotic Jurkat T cells and increased gradually up to 6 h, and slightly declined at 12 h, but at 24 h the level of COX-2 mRNA declined (Figure 1(a)). In contrast, viable Jurkat cells did not affect COX-2 mRNA expression over this time period (Figure 1(b)). There was no change in COX-1 mRNA expression within 24 h of exposure to apoptotic or viable Jurkat cells (Figure 1(a)). In addition, COX-2 mRNA expression was also measured following exposure to various cell types. Exposure to apoptotic neutrophils, apoptotic HeLa cells, and apoptotic thymocytes also induced COX-2 mRNA expression, but the timing of peak expression differed (Figures 1(c)–1(e)). The peak increase in COX-2 mRNA expression was observed at 1, 2, and 8 h after exposure to apoptotic HeLa cells, neutrophils, and thymocytes, respectively. Why the kinetics of COX-2 mRNA expression are different is not clearly explained in this experimental setting, but different cell types may cause that.

We analyzed the levels of COX-2 mRNA expression following exposure to UV-irradiated apoptotic and aged apoptotic human neutrophils after standardization of the amount of these apoptotic cells, since the proportion of apoptotic cells in aged neutrophils is lower compared to UV-irradiated neutrophils (61% positive for apoptotic aged neutrophils as detected by Annexin V staining versus 80% positive for UV-irradiated apoptotic neutrophils) [16]. In addition to UV-irradiated apoptotic cells, aged apoptotic human neutrophils induced significantly COX-2 mRNA expression (Figure 1(c)). These findings suggest that COX-2 mRNA expression induced by exposure to apoptotic cells in macrophages is a global phenomenon independent of cell type and apoptotic process.

Expression of COX-1 and COX-2 protein was evaluated by immunoblot analysis of lysates of cultured RAW 264.7 cells. COX-2 expression increased progressively up to 24 h after addition of apoptotic Jurkat cells, but COX-1 expression did not change over this time period (Figure 1(f)). Exposure to viable cells had no effect on either COX-2 or COX-1 expression over the period examined (Figure 1(g)). Pretreatment of RAW 264.7 cells with either actinomycin D or cycloheximide for 1 h before stimulation with apoptotic cells completely inhibited COX-2 expression, indicating that COX-2 mRNA* de novo* synthesis is required for its protein expression (Figure 1(h)). Confocal microscopy also demonstrated progressive increase in COX-2 protein in RAW 264.7 cells from 2 to 24 h after* in vitro* exposure to apoptotic cells (Figure 1(i)).

### 3.2. *In Vitro* Exposure of Macrophages to Apoptotic Cells Induces COX-2-Dependent PGE_2_ Production

**Figure 2:**
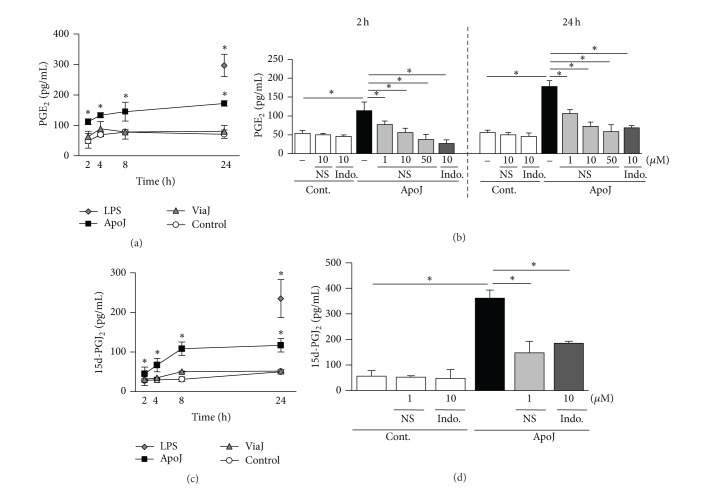
Apoptotic cells induce PGE_2_ and 15d-PGJ_2 _production by RAW 264.7 cells. (a, c) RAW 264.7 cells were stimulated by apoptotic (ApoJ), viable (ViaJ) Jurkat T cells, or lipopolysaccharide (LPS) for the times indicated. (b, d) After 1 h pretreatment with 1–50 *μ*M of NS-398 or 10 *μ*M of indomethacin (Indo), RAW cells were stimulated with ApoJ for 2 h or 24 h. PGE_2_ or 15d-PGJ_2_ levels in the conditioned media were measured by EIA. Values represent mean ± SEM of three or more separate experiments, *P < 0.05, compared with control or ApoJ versus ApoJ + NS or Indo.

PGE_2_ secretion, as measured by EIA, increased significantly in RAW 264.7 cells following exposure to apoptotic Jurkat cells (Figure 2(a)). A significant increase in PGE_2_ production was observed 2 h after* in vitro* exposure to apoptotic cells, and PGE_2_ production continued to increase up to 24 h. To confirm that COX-2 induction by exposure to apoptotic cells mediates the enhanced PGE_2_ production in macrophages, RAW 264.7 cells were pretreated with the highly selective COX-2 inhibitor NS-398 (1~50 *μ*M) or the nonselective COX inhibitor indomethacin (10 *μ*M) and incubated with apoptotic Jurkat T cells for 2 or 24 h. NS-398 reduced apoptotic cell-induced PGE_2_ secretion in a dose-dependent manner (Figure 2(b)). Indomethacin also inhibited apoptotic cell-induced PGE_2_ production. These data suggest that the apoptotic cell-induced increase in PGE_2_ production in RAW 264.7 cells derives predominantly from induction of COX-2 expression. The expression of another product of the COX-2 pathway, 15d-PGJ_2_, was similarly enhanced (Figure 2(c)). Apoptotic cell-induced 15d-PGJ_2_ secretion was also reduced by 1 *μ*M NS-398 or 10 *μ*M indomethacin (Figure 2(d)). These data suggest that the apoptotic cell-induced increase in PGE_2_ and 15d-PGJ_2_ production in RAW 264.7 cells derives predominantly from induction of COX-2 expression.

### 3.3. Enhancement of COX-2/PGE_2_ Signaling by Interaction with Apoptotic Cells Mediates the Upregulation of HGF Production

**Figure 3:**
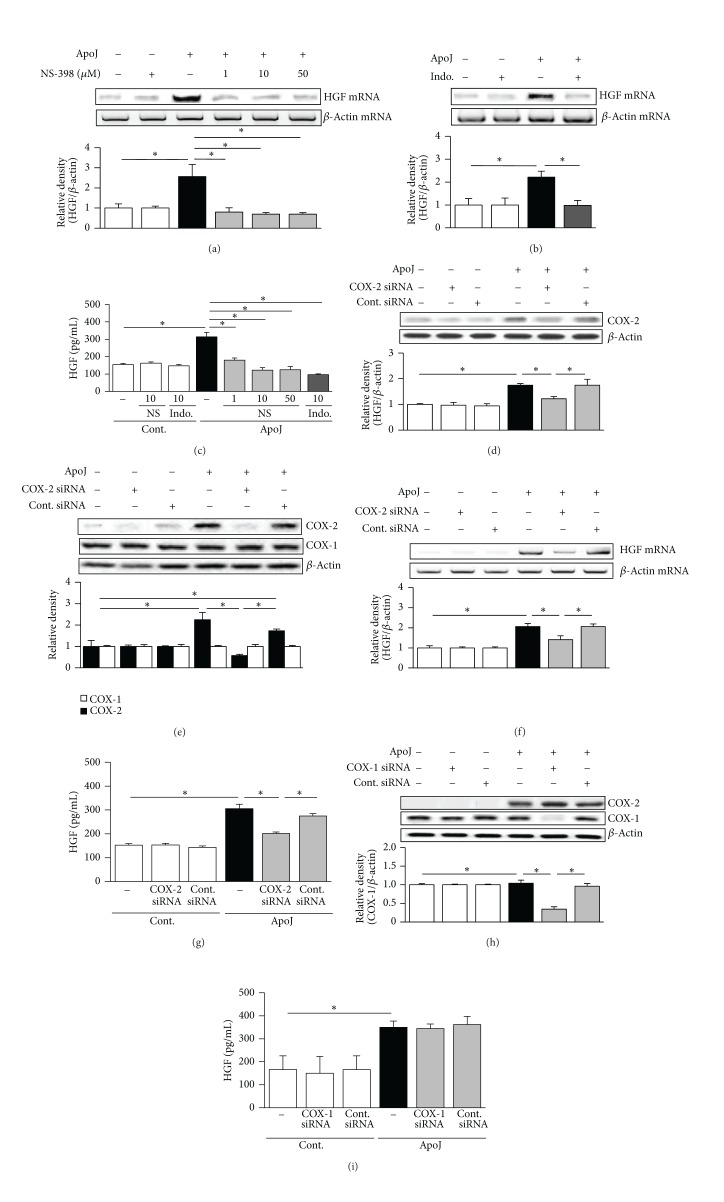
Inhibition of COX-2 downregulates HGF expression. RAW 264.7 cells were pretreated with NS-398 or indomethacin (Indo) for 1 h and then stimulated with apoptotic Jurkat cells (ApoJ) for 2 h to detect HGF mRNA expression (a, b) or for 24 h to detect secreted HGF (c). RAW cells were transfected with COX-2 or control vehicle (siRNA-GFP) for 6 h and then incubated with ApoJ for 2 (d, f) or 24 h (e, g). (h, i) RAW cells were transfected with COX-1 siRNA or control vehicle (siRNA-GFP) for 48 h and then incubated with ApoJ for 24 h. (a, b, c) HGF mRNA levels were analyzed using semiquantitative RT-PCR and normalized to *β*-actin mRNA levels. (d, e, h) Immunoblots of total cell lysates were performed with anti-COX-2 or COX-1 antibodies. (c, g, i) HGF levels in conditioned media were measured by ELISA. Values represent mean ± SEM of three or more separate experiments; *P < 0.05.

Park et al. [4] reported that enhanced HGF mRNA expression in RAW 264.7 cells following apoptotic cell exposure peaks at 2 h and that secretion of HGF protein is increased 24 h after exposure. In the present study, the role of COX-2 in apoptotic cell-induced HGF expression was also evaluated at these time points. Experiments were performed using pharmacologic inhibitors, such as NS-398, indomethacin, and siRNAs targeting COX-2 or COX-1. RAW 264.7 cells were pretreated with NS-398 or indomethacin and were then cultured with apoptotic Jurkat cells for 2 h to assess the effect on HGF mRNA expression and 24 h to assess the effect on HGF protein production. NS-398 (1, 10, and 50 *μ*M) and indomethacin (10 *μ*M) completely inhibited apoptotic cell-induced HGF mRNA (Figures 3(a) and 3(b)). Complete inhibition of HGF protein expression was also shown by treatment with 10 and 50 *μ*M NS-398 and 10 *μ*M indomethacin (Figure 3(c)).

To further examine the contribution of COX-2 to apoptotic cell-induced HGF expression in RAW 264.7 cells, experiments were performed using COX-2-specific siRNA. The negative control siRNA did not alter the COX-2 protein level in cells stimulated with apoptotic cells. COX-2 protein expression was completely inhibited at 2 and 24 h after apoptotic cell exposure in cells transfected with COX-2 siRNA by 67 and 100%, respectively, (Figures 3(d) and 3(e)) but COX-1 protein levels were unchanged, as determined by immunoblot analysis. Knockdown of the COX-2 gene prevented apoptotic cell-induced HGF mRNA and protein expression without affecting the mRNA and protein expression of the endogenous control, *β*-actin (Figures 3(f) and 3(g)). In contrast, when COX-1 expression was silenced by transfection with COX-1-specific siRNA, HGF protein secretion was unaffected (Figures 3(h) and 3(i)). These data strongly suggest that only COX-2 induction is required for the induction of HGF mRNA and protein expression in RAW cells exposed to apoptotic cells* in vitro*.

**Figure 4:**
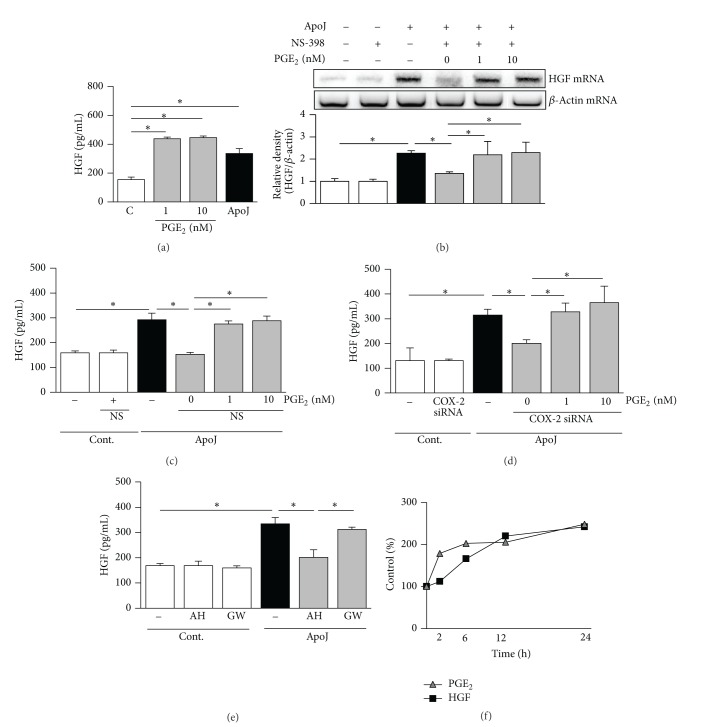
COX-2/PGE_2_ signaling is required for HGF production in response to apoptotic cells. (a) RAW 264.7 cells were treated with 1 or 10 nM of PGE_2_ for 24 h to detect secreted HGF. (b, c) After 1 h pretreatment of 10 *μ*M NS-398, apoptotic Jurkat cells (ApoJ) with or without PGE_2_ were added to RAW cells for 2 h to detect HGF mRNA or for 24 h to detect secreted HGF. (d) Transfected RAW cells with siRNA against COX-2 were stimulated with ApoJ with or without 1 or 10 nM PGE_2_ for 24 h. (e) Raw cells were pretreated with 10 *μ*M of EP2 receptor antagonist, AH-6809 (AH), or 10 *μ*M of EP4 receptor antagonist, GW 627368X (GW), for 1 h and then stimulated with ApoJ for 24 h. (a, c–e) HGF levels in conditioned media were measured by ELISA. (b) HGF mRNA levels were analyzed by semiquantitative RT-PCR. Values represent mean ± SEM of three or more separate experiments; *P < 0.05. (f) RAW cells were stimulated with ApoJ for a 24 h period and PGE_2_ and HGF levels in the supernatants were measured at each time points by EIA and ELISA, respectively, and expressed as percent of control. Data are representative from separate three experiments.

Treatment of RAW 264.7 cells with 1 or 10 nM PGE_2_ resulted in increases in the level of HGF protein in the culture medium (Figure 4(a)). We then examined the involvement of PGE_2_ in mediating the effects of COX-2 on HGF induction. RAW 264.7 cells were treated with PGE_2_ together with apoptotic cells in the presence of 10 *μ*M NS-398. Addition of 1 or 10 nM PGE_2_ completely restored HGF mRNA expression suppressed by COX-2 inhibition (Figure 4(b)). Similarly, the reduced HGF secretion by cells treated with NS-398 or transfected with COX-2-specific siRNA was completely restored by addition of PGE_2_ (Figures 4(c) and 4(d)).

A number of PGE_2_-specific receptors have been identified, including EP1, EP2, EP3, and EP4 [17, 18], and it has been reported that macrophages can express both EP2 and EP4 [19]. To determine which receptors are involved in the apoptotic cell-induced PGE_2_ signaling pathway, RAW 264.7 cells were pretreated with the EP2 receptor antagonist AH-6809 or the EP4 receptor antagonist GW-627368X for 1 h before apoptotic cells were added. The EP2 receptor antagonist, but not the EP4 receptor antagonist, blocked apoptotic cell-induced HGF secretion (Figure 4(e)). When RAW 264.7 cells were incubated with apoptotic Jurkat cells over a 24 h period, kinetic analysis showed that the rise in PGE_2_ preceded the increase in HGF production (Figure 4(f)). Regarding the effects of COX-2 inhibition using pharmacologic and genetic approaches, these results demonstrate that the COX-2/PGE_2_/EP2 axis induced by apoptotic cell exposure is instrumental in upregulating HGF production.

### 3.4. COX-2/PGE_2_ Signaling Is Required for Upregulation of HGF Expression in Murine Peritoneal Macrophages in Response to Apoptotic Cells

**Figure 5:**
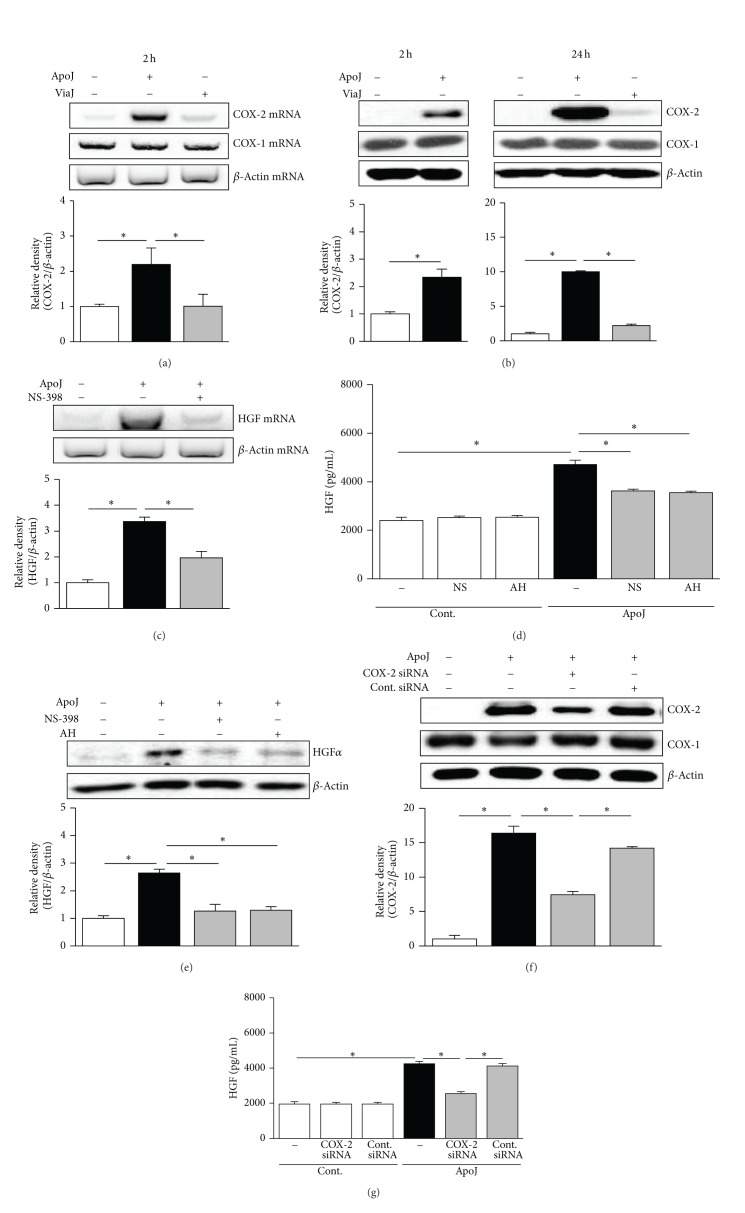
Apoptotic cell-induced HGF production in murine peritoneal macrophages is dependent on COX-2/PGE_2_ signaling. (a, b) Murine peritoneal macrophages were stimulated with apoptotic (ApoJ) or viable (ViaJ) Jurkat cells for the times indicated. After 1 h of pretreatment of 10 *μ*M NS-398 or 10 *μ*M AH-6809 (AH), peritoneal macrophages were stimulated with ApoJ for 2 h to detect HGF mRNA (c) or for 24 h to detect HGF protein (d, e). (f, g) Peritoneal macrophages were transfected with COX-2 siRNA or control vehicle (siRNA-GFP) for 6 h and then stimulated with ApoJ for 24 h. (a, c) COX-2, COX-1, or HGF mRNA levels were analyzed using semiquantitative RT-PCR and normalized to *β*-actin mRNA levels. (b, e, f) Immunoblots of total cell lysates were performed with anti-COX-2, COX-1, or HGF*α* antibodies. (d, g) HGF levels in conditioned media were measured by ELISA. Values represent mean ± SEM of three or more separate experiments; *P < 0.05.

In addition to RAW 264.7 macrophages, we also examined COX-2/PGE_2_ signaling in a primary cell model by isolating resident peritoneal macrophages by lavage from naive mice and then incubating these cells with apoptotic or viable Jurkat T cells. Apoptotic cell stimulation resulted in increased COX-2 mRNA and protein expression by the peritoneal macrophages, whereas exposure to viable cells did not (Figures 5(a) and 5(b)). There were no changes in COX-1 mRNA and protein expression. HGF mRNA expression induced by apoptotic cell exposure was inhibited by pretreatment with 10 *μ*M NS-398 (Figure 5(c)). Furthermore, inhibition of COX-2 mRNA expression with NS-398 (10 *μ*M) and inhibition of the EP2 receptor with AH-6809 (10 *μ*M) significantly reduced HGF secretion (Figure 5(d)). Immunoblot analysis of peritoneal macrophage lysates using anti-HGF *α*-chain antibody indicated that intracellular HGF protein expression was also reduced by treatment with NS-398 or AH-6809 (Figure 5(e)).

To confirm that COX-2 is required for apoptotic cell-induced HGF expression, peritoneal macrophages were transfected with COX-2-specific siRNA or a negative control siRNA, before addition of apoptotic Jurkat cells. Apoptotic cell-induced COX-2 protein expression decreased by approximately 60% in cells transfected with COX-2-specific siRNA, but COX-1 protein expression did not change (Figure 5(f)). Upon exposure of primary peritoneal macrophages to apoptotic cells, the siRNA-mediated silencing of COX-2 mRNA expression significantly inhibited HGF production (Figure 5(g)), as was observed in RAW 264.7 cells.

### 3.5. HGF Activation Mediates Upregulation of COX-2/PGE_2_ in RAW 264.7 and Primary Peritoneal Macrophages in Response to Apoptotic Cell Exposure

**Figure 6:**
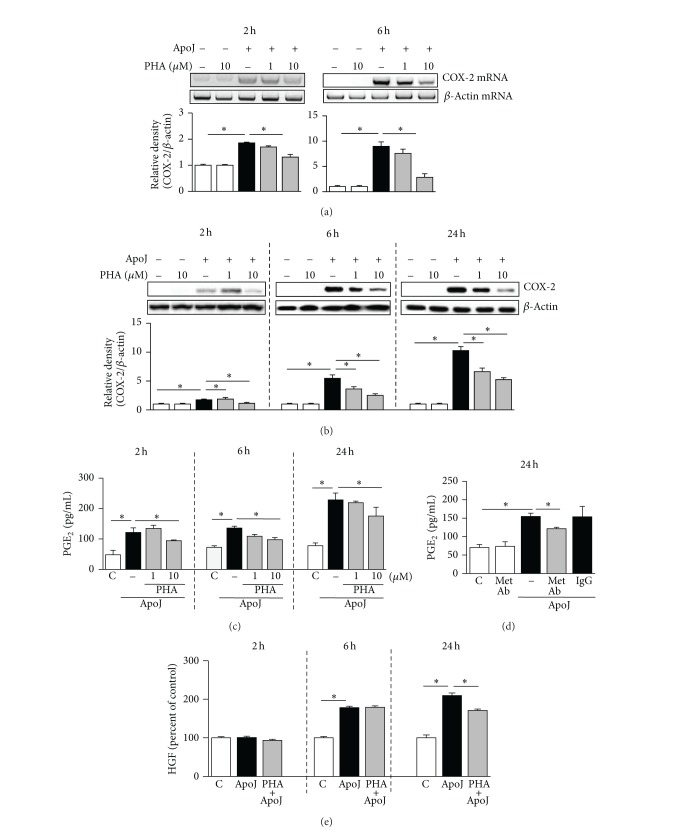
HGF receptor antagonist suppresses COX-2 expression and PGE_2_ production in RAW 264.7 cells in response to apoptotic cells. RAW 264.7 cells were pretreated with PHA-665752 (1 or 10 *μ*M), c-Met antibody (10 *μ*g/mL), or IgG (10 *μ*g/mL) for 1 h and then stimulated with apoptotic Jurkat cells (ApoJ) for the times indicated. (a) COX-2 mRNA levels were analyzed by semiquantitative RT-PCR. Total cell lysates were immunoblotted for COX-2 (b) and cell supernatants were measured for PGE_2_ (c, d) by EIA or HGF by ELISA (e). Values represent mean ± SEM of three or more separate experiments; *P < 0.05.

A previous report suggested that COX-2 and PGE_2_ are downstream mediators of HGF expression in fibroblasts [9], which encouraged us to investigate the effect of HGF in mediating* in vitro* apoptotic cell-induced COX-2 and PGE_2_ expression by macrophages. We evaluated the effect of PHA-665752, a selective inhibitor of the HGF receptor c-Met, on COX-2 mRNA and protein expression. At 2 and 6 h after* in vitro* exposure to apoptotic Jurkat cells, COX-2 mRNA expression was significantly inhibited by 10 *μ*M PHA-665752 but not by 1 *μ*M PHA-665752 (Figure 6(a)). COX-2 protein expression was significantly inhibited in a dose-dependent manner by 1 and 10 *μ*M PHA-665752 at 6 and 24 h after* in vitro* exposure to apoptotic cells (Figure 6(b)). Similarly, the pattern of PGE_2_ production after inhibition of HGF signaling paralleled that of COX-2 protein expression. At 6 and 24 h after* in vitro* exposure to apoptotic cells, PGE_2_ secretion was suppressed by both 1 and 10 *μ*M PHA-665752 in a dose-dependent manner (Figure 6(c)). To confirm the effect of HGF on PGE_2_ production in RAW 264.7 cells, c-Met blocking antibody was also used. This antibody suppressed PGE_2_ secretion by approximately 40% at 24 h after exposure to apoptotic cells (Figure 6(d)). Furthermore, 10 *μ*M PHA-665752 decreased partially HGF production at 24 h after* in vitro* exposure to apoptotic Jurkat cells, implicating the possibility of a positive feedback loop between COX-2/PGE_2_ and HGF signaling pathways (Figure 6(e)).

**Figure 7:**
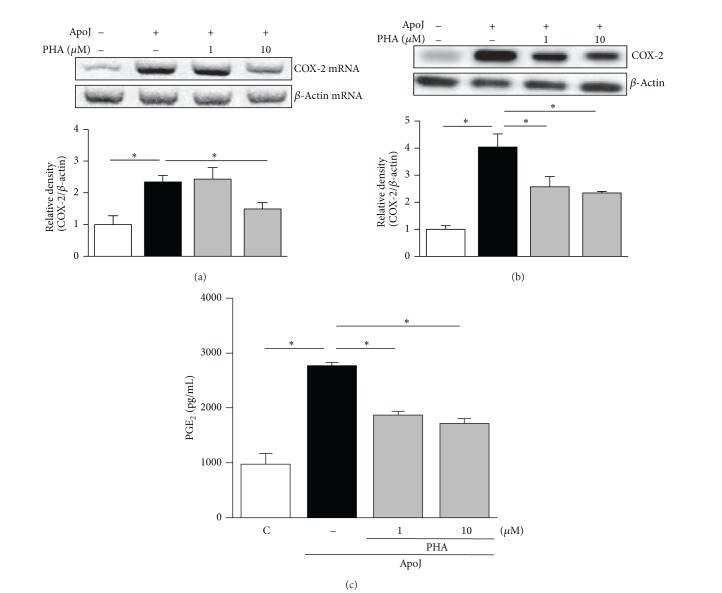
HGF receptor antagonist suppresses COX-2 expression and PGE_2_ production in peritoneal macrophages in response to apoptotic cells. Murine peritoneal macrophages were pretreated with PHA-665752 (1 or 10 *μ*M) for 1 h and then stimulated with apoptotic Jurkat cells (ApoJ). (a) At 2 h after* in vitro* exposure to ApoJ, COX-2 mRNA levels were analyzed by semiquantitative RT-PCR. At 24 h after* in vitro* exposure to ApoJ, total cell lysates were immunoblotted for COX-2 (b) and cell supernatants were measured for PGE_2_ by EIA (c). Values represent mean ± SEM of three or more separate experiments; *P < 0.05.

The involvement of HGF signaling in enhancing induction of COX-2 and PGE_2_ expression in response to apoptotic cell exposure was also shown in primary murine peritoneal macrophages, in which 1 or 10 *μ*M PHA-665752 inhibited COX-2 mRNA and protein expression as well as PGE_2_ production (Figures 7(a)–7(c)). The suppressive effects of PHA-665752 on COX-2 mRNA and protein expression as well as PGE_2_ production were consistent and dose-dependent, indicating that HGF partially mediates COX-2 expression and PGE_2_ production induced by apoptotic cells.

### 3.6. COX-2/PGE_2_ Signaling Is Not Required for Upregulation of TGF-*β* in Unstimulated Macrophages in Response to Apoptotic Cell Exposure

**Figure 8:**
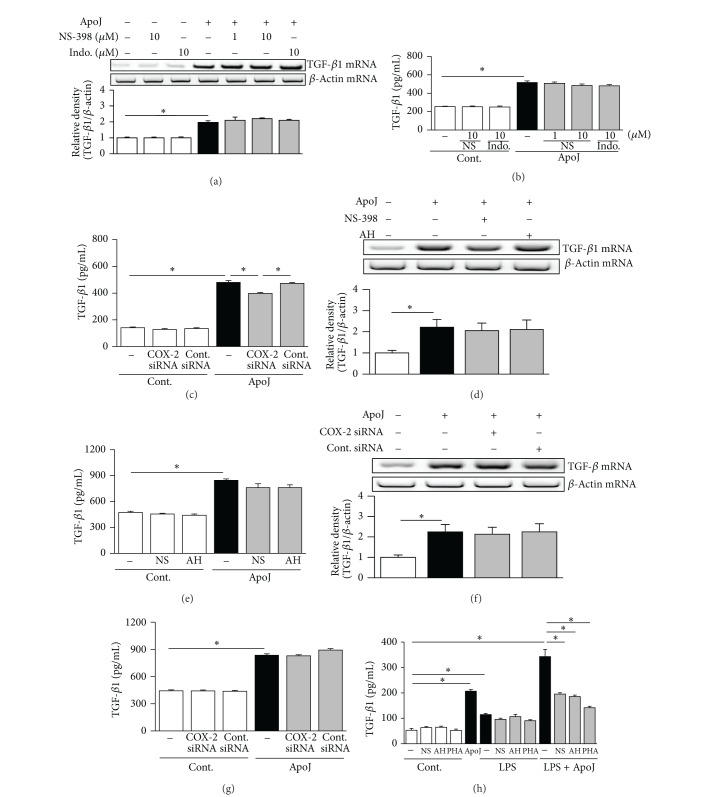
TGF-*β*1 induction in unstimulated macrophages in response to apoptotic cells is not notably suppressed by COX-2 inhibition. RAW 264.7 cells (a, b) or peritoneal macrophages (d, e) were pretreated with NS-398, indomethacin (Indo), or AH-6809 (AH) for 1 h and then stimulated with apoptotic Jurkat cells (ApoJ) for 2 h to detect TGF-*β*1 mRNA expression (a, d) or for 24 h to detect secreted TGF-*β*1 (b, e). RAW cells (c) or peritoneal macrophages (f, g) were transfected with COX-2 siRNA or control vehicle (siRNA-GFP) for 6 h and then stimulated with ApoJ for 2 h to detect TGF-*β*1 mRNA expression, or for 24 h to detect secreted TGF-*β*1. (h) Raw cells were pretreated with 10 *μ*M NS-398, 10 *μ*M AH-6809, or 10 *μ*M PHA-665752 for 1 h and then stimulated with 1 *μ*g/mL LPS and ApoJ. (a, d, f) TGF-*β*1 mRNA levels were analyzed using semiquantitative RT-PCR and normalized to *β*-actin mRNA levels. (b, c, e, g, h) TGF-*β*1 levels in conditioned media were measured by ELISA. Values represent mean ± SEM of three or more separate experiments; *P < 0.05.

TGF-*β* expression is also induced by apoptotic cells [1] but is reciprocally balanced with HGF [20]. Different regulatory systems are thought to be involved in the induction of HGF and TGF-*β* expression. Thus, we examined whether COX-2/PGE_2_ signaling functions differently in TGF-*β*1 production following apoptotic cell exposure. Neither NS-398 nor indomethacin reduced apoptotic cell-induced TGF-*β*1 mRNA expression in RAW 264.7 cells (Figure 8(a)). Similarly, TGF-*β*1 protein secretion did not decrease significantly following COX-2 inhibition (Figure 8(b)). COX-2-specific siRNA resulted in limited inhibition of TGF-*β*1 secretion (24% reduction) (Figure 8(c)). Compared to the effect on HGF expression, which was suppressed to the basal level following COX-2 inhibition, the reduction in TGF-*β*1 secretion was not remarkable. In resident peritoneal macrophages from naive mice, apoptotic cell-induced TGF-*β*1 mRNA and protein expression were unaffected or only minimally decreased by pharmacologic inhibition of COX-2 and PGE_2_ signaling using 10 *μ*M NS-398 and 10 *μ*M AH-6809 (Figures 8(d) and 8(e)). Similarly, COX-2-specific siRNA did not inhibit induction of TGF-*β*1 mRNA and protein expression in peritoneal macrophages (Figures 8(f) and 8(g)). These findings demonstrate that COX-2/PGE_2_ signaling is not required for apoptotic cell-induced upregulation of TGF-*β*1 mRNA and protein expression in macrophages.

Data from previous and present studies provide evidence that COX-2/PGE_2_ and HGF activation are not involved in TGF-*β* production in unstimulated RAW 264.7 cells in response to apoptotic cells [11]. Therefore, we wondered whether COX-2/PGE_2_ and HGF activation mediate TGF-*β* production in stimulated RAW 264.7 cells in response to apoptotic cells. Macrophages were treated with 1.0 *μ*g/mL LPS; at the same time, apoptotic cells were added. Similar to the findings of Fadok and colleagues [1], the levels of TGF-*β* by LPS-stimulated macrophages were enhanced significantly in response to apoptotic cells when compared with LPS-treated macrophages (Figure 8(h)). By adding the effect of pharmacologic inhibitors, including NS-398, AH-6809, or PHA-665752, TGF-*β* production in LPS-stimulated macrophages was significantly inhibited at 24 h after exposure to apoptotic cells. These data indicate that anti-inflammatory response after apoptotic cell recognition is mediated, at least in part, via TGF-*β* derived from the positive cross talk between COX-2/PGE_2_/EP2 and HGF/c-Met signaling pathways.

## 4. Discussion

The present study adds to the emerging view that macrophages recognizing apoptotic cells themselves can reinforce signaling pathways toward greater production of anti-inflammatory and antifibrotic mediators in a feedforward manner. We first demonstrated that expression of COX-2 (but not COX-1) mRNA and protein increased in RAW 264.7 cells as well as primary peritoneal macrophages exposed to apoptotic Jurkat T cells. The COX-2 mRNA induction was also observed when RAW 264.7 cells were exposed to other types of apoptotic cells, such as those of human neutrophils, HeLa epithelial cells, murine thymocytes, indicating the universality of the phenomenon independent of cell type. In addition to UV-irradiated apoptotic cells, aged apoptotic human neutrophils also induced COX-2 mRNA expression. These data suggest that macrophages have switched their functional program in response to dying cells and triggered the COX-2-dependent pathways for production of immunosuppressive and/or regenerative mediators.

Various PGs are autocrine mediators derived from metabolism of arachidonate through the action of COX. In this study we focused on PGE_2_ synthesis because of its physiologic effects, which include limitation of the immune-inflammatory response and control of tissue repair processes.* In vitro* exposure of RAW 264.7 cells to apoptotic Jurkat cells increased PGE_2_ production. Moreover, our time course study showed rapid induction and a continuous increase in PGE_2_ production up to 24 h. Previous reports suggested that increased PGE_2_ production along with decreased expression of proinflammatory eicosanoids, thromboxane B_2_, and leukotriene C_4_ in human monocyte-derived macrophages in response to apoptotic cell exposure is indicative of a selective effect of apoptotic cell uptake on macrophage eicosanoid generation [1]. In the present study, using the highly selective COX-2 inhibitor NS-398, we provide evidence that PGE_2_ production in macrophages is enhanced by exposure to apoptotic cells, predominantly via induction of COX-2 expression.

Pharmacologic inhibition of COX-2 activity and siRNA-mediated knockdown of COX-2 expression reduced apoptotic cell-induced HGF mRNA and protein expression in RAW 264.7 cells and primary peritoneal macrophages. However, knockdown of COX-1 expression did not affect HGF production in response to apoptotic cell exposure. These findings suggest that COX-2, but not COX-1, is specifically involved in the production of HGF. Addition of PGE_2_ restored HGF mRNA and protein expression, which were suppressed by pharmacologic inhibition or genetic knockdown of COX-2 activity or expression. These data provide evidence that the effects of COX-2 on HGF induction in response to apoptotic cells are mediated by a direct effect of COX-2-derived PGE_2_ on macrophages. Moreover, inhibition of PGE_2_ signaling by an EP2 receptor antagonist (but not an EP4 antagonist) suppressed apoptotic cell-induced HGF production. Transcription of the HGF gene and HGF production are well known to be stimulated by substances that increase cyclic AMP, including PGE_2_ [21, 22]. It is therefore likely that COX-2-derived PGE_2_ induces transcriptional HGF production by macrophages in response to apoptotic cells via the cyclic AMP pathway after interaction with an EP2 receptor. However, the COX-2/PGE_2_/EP2 axis is not the only signaling pathway involved in apoptotic cell-induced HGF production. A series of experiments in a previous study emphasized the importance of the RhoA/Rho kinase/PI3K/Akt/MAPK (including p38 MAPK, ERK, and JNK) axis, which is required for the upregulation of HGF mRNA and protein expression in RAW 264.7 cells in response to apoptotic cell exposure [8]. The PI3K/Akt and MAPK pathways also reportedly play a role in the regulation of COX-2 expression in response to a variety of extracellular stimuli [23]. Following MAPK signaling, the activation of transcriptional factors such as E-26 like protein 1 (ELK-1), activating transcription factor 2 (ATF-2), STAT, c-fos, c-Jun, and AP-1 in the COX-2 promoter region can increase COX-2 expression [24]. Our data indicate that RhoA is not involved in apoptotic cell-induced COX-2 expression because neither knockdown of RhoA nor inhibition of RhoA with the specific inhibitor C3 transferase suppressed COX-2 protein production (data not shown). However, the role of the PI3K/Akt and MAPK pathways in COX-2 induction by apoptotic cells requires further investigation.

Likewise, production of another COX-2-derived PG, 15d-PGJ_2_, was also enhanced by* in vitro* exposure of macrophages to apoptotic Jurkat cells. Indeed,* in vitro* exposure of macrophages to apoptotic cells has been shown to increase the intracellular levels of PGE synthase1 as well as PGD synthase [2]. Similar to PGE_2_, 15d-PGJ_2_ secretion in response to apoptotic cells was derived predominantly via induction of COX-2 expression. In addition, 15d-PGJ_2_ induces expression of HGF in mesangial cells via a peroxisome proliferator response element in the HGF promoter through activation of peroxisome proliferator-activated receptors (PPAR)-*γ*. Here, we demonstrated 15d-PGJ_2_-induced HGF production in macrophages (data not shown). Thus, 15d-PGJ_2_ may also participate in the upregulation of HGF expression by macrophages exposed to apoptotic cells, although this effect was not examined in our current study.

A number of prosurvival factors, including HGF and COX-2/PGE_2_, normally promote survival of epithelial and endothelial cells, fibroblast quiescence, and normal regulation of the extracellular matrix [25]. HGF signaling via the Met receptor upregulates COX-2 expression in different cell types, including fibroblasts and epithelial cells [26], and animal models have revealed a role for HGF as a mediator of COX-2/PGE_2_ signaling-driven antifibrosis* in vivo* [9]. Thus, we investigated the possibility of cross talk between the HGF and COX-2/PGE_2_ signaling pathways by exposing RAW 264.7 cells and peritoneal macrophages to apoptotic cells* in vitro*. The c-Met inhibitor PHA-665752 suppressed expression of COX-2 mRNA and protein in macrophages in response to apoptotic cells. Similarly, this inhibitor reduced PGE_2_ production in a dose-dependent manner. Based on data from the time course study, decrease in PGE_2_ production in RAW 264.7 cells was minimal at 24 h (~35% reduction) and maximum at 6 h (~60% reduction) after exposure to apoptotic cells. Using another c-Met inhibitor, a blocking antibody, we found a similar effect on PGE_2_ production at 24 h (~40% reduction). These data suggest that HGF/c-Met signaling alone does not fully exert positive effect on synthesis of PGE_2_ by exposure to apoptotic cells. Moreover, PHA-665752 also reduced partially HGF production after* in vitro* exposure to apoptotic Jurkat cells. Collectively, our data suggest that the HGF/c-Met and COX-2/PGE_2_ signaling pathways are interrelated through positive cross talk in macrophages stimulated by apoptotic cells over a 24 h period. This positive feedback loop may provide, at least in part, greater PGE_2_ and HGF production in macrophages, which contribute to anti-inflammatory and antifibrotic activities induced by the interaction with apoptotic cells [12]. However, other mediators are likely involved and might participate in COX-2 and PGE_2_ expression through different mechanisms since the c-Met inhibitor inhibited in part the PGE_2_ as well as HGF production. In studies by Freire-de-Lima and colleagues [2], arachidonic acid release, COX-2 protein expression, and PGE_2_ production are significantly dependent on the TGF-*β* production in response to apoptotic cells. Indeed, macrophages expressing the truncated TGF-*β* receptor did not show upregulation of COX-2 and PGE_2_ in response to apoptotic cells. Thus, although not explored directly herein, it seems reasonable to assume that released TGF-*β* contributes to greater COX-2 and PGE_2_ expression, leading to HGF production.

Even though TGF-*β* has been shown to play pivotal roles in the anti-inflammatory and anti-immunogenic responses to apoptotic cell clearance, it often acts as an antiproliferative or profibrotic agent. In contrast, HGF is an important tissue repair molecule. Administration of exogenous HGF accelerates tissue repair in several organs after acute injury or ischemia [27, 28]. TGF-*β* is a potent negative regulator of HGF expression [29], and HGF seems to have biological activities that oppose those of TGF-*β* through diverse mechanisms [26]. Notably, our data indicate that COX-2/PGE_2_ signaling is involved in transcription of HGF but not TGF-*β*1 in unstimulated macrophages in response to apoptotic cells. In addition, data from previous studies using anti-c-Met blocking antibody showed that HGF activation is not involved in TGF-*β* production in unstimulated RAW 264.7 cells in response to apoptotic cells [11]. However, in LPS-stimulated macrophages, our* in vitro* studies using pharmacological inhibitors provide evidence that COX-2/PGE_2_ and HGF signaling pathways are involved in enhancement of TGF-*β* production in response to apoptotic cells. Thus, in this limited context, we see the possibility that anti-inflammatory response after apoptotic cell recognition is mediated, at least in part, via TGF-*β* derived from the positive cross talk between COX-2/PGE_2_/EP2 and HGF/c-Met signaling pathways in the inflammatory lesion. On the other hand, prolonged inhibition of the COX-2/PGE_2_ or HGF signaling pathway reversed the reduction of TGF-*β*1 production and the hydroxyproline content in lung tissue following* in vivo* exposure to apoptotic cells at the late fibrotic phase [12]. Thus, the COX-2/PGE_2_ and HGF signaling pathways may provide an important additional control of the balance between HGF and TGF-*β*, favoring antifibrotic effects in the efferocytosis system.

**Figure 9:**
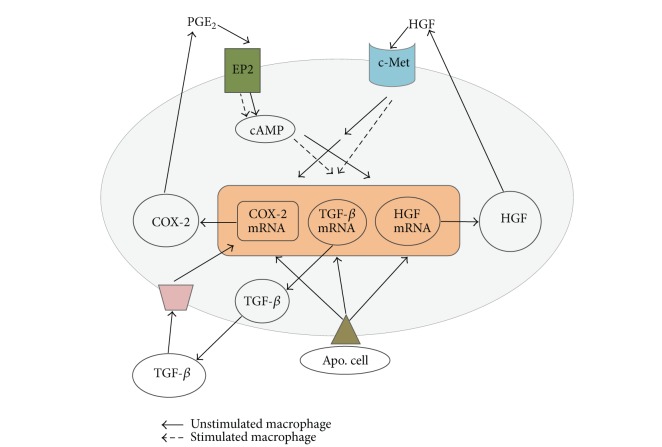
A schematic diagram proposed COX-2/PGE_2_ and HGF pathways for a positive feedback loop associated with production of anti-inflammatory cytokine TGF-*β* in response to apoptotic cells.

In conclusion, the findings of this study reveal a novel function of COX-2/PGE_2_/EP2 signaling in macrophages exposed to apoptotic cells* in vitro* that results in enhanced HGF expression. Furthermore, the COX-2/PGE_2_ pathway showed different regulatory effects for TGF-*β*1, and HGF activation was shown to mediate COX-2 and PGE_2_ expression induced by exposure to apoptotic cells* in vitro*. Therefore, it is likely that the ability of COX-2/PGE_2_/EP2 signaling to promote HGF synthesis is part of a positive feedback loop that results in amplification of macrophage COX-2 and PGE_2_ expression (Figure 9). This positive cross talk mechanism between COX-2/PGE_2_/EP2 and HGF/c-Met signaling pathways may contribute to the anti-inflammatory and antifibrotic consequences of apoptotic cell recognition.
